# Combined treatment with Sigma1R and A2AR agonists fails to inhibit cocaine self-administration despite causing strong antagonistic accumbal A2AR-D2R complex interactions: the potential role of astrocytes

**DOI:** 10.3389/fnmol.2023.1106765

**Published:** 2023-05-24

**Authors:** Dasiel O. Borroto-Escuela, Alexander Lopez-Salas, Karolina Wydra, Marco Bartolini, Zilong Zhou, Malgorzata Frankowska, Agata Suder, Javier Benitez-Porres, Wilber Romero-Fernandez, Malgorzata Filip, Kjell Fuxe

**Affiliations:** ^1^Department of Neuroscience, Karolinska Institutet, Stockholm, Sweden; ^2^Department of Human Physiology, Physical Education and Sport, Universidad de Málaga, Málaga, Spain; ^3^Department of Biomolecular Science, Section of Physiology, University of Urbino, Urbino, Italy; ^4^Maj Institute of Pharmacology Polish Academy of Sciences, Department of Drug Addiction Pharmacology, Kraków, Poland; ^5^Department of Medical Biotechnology and Translational Medicine, University of Milan, Milan, Italy; ^6^National Engineering Laboratory for Druggable Gene and Protein Screening, Northeast Normal University, Changchun, China; ^7^Department of Neurology, Vanderbilt University Medical Center, Nashville, TN, United States

**Keywords:** A2AR-D2R heteroreceptor complexes, allosteric receptor-receptor interactions, cocaine use disorder, monoamine stabilizer, G protein coupled receptor (GPCR), oligomerization, Sigma 1 receptor

## Abstract

Previous studies have indicated that acute treatment with the monoamine stabilizer OSU-6162 (5 mg/kg), which has a high affinity for Sigma1R, significantly increased the density of accumbal shell D2R-Sigma1R and A2AR-D2R heteroreceptor complexes following cocaine self-administration. *Ex vivo* studies using the A2AR agonist CGS21680 also suggested the existence of enhanced antagonistic accumbal A2AR-D2R allosteric interactions after treatment with OSU-6162 during cocaine self-administration. However, a 3-day treatment with OSU-6162 (5 mg/kg) failed to alter the behavioral effects of cocaine self-administration. To test these results and the relevance of OSU-6162 (2.5 mg/kg) and/or A2AR (0.05 mg/kg) agonist interactions, we administered low doses of receptor agonists during cocaine self-administration and assessed their neurochemical and behavioral effects. No effects were observed on cocaine self-administration; however, marked and highly significant increases using the proximity ligation assay (PLA) were induced by the co-treatment on the density of the A2AR-D2R heterocomplexes in the nucleus accumbens shell. Significant decreases in the affinity of the D2R high- and low-affinity agonist binding sites were also observed. Thus, in low doses, the highly significant neurochemical effects observed upon cotreatment with an A2AR agonist and a Sigma1R ligand on the A2AR-D2R heterocomplexes and their enhancement of allosteric inhibition of D2R high-affinity binding are not linked to the modulation of cocaine self-administration. The explanation may be related to an increased release of ATP and adenosine from astrocytes in the nucleus accumbens shell in cocaine self-administration. This can lead to increased activation of the A1R protomer in a putative A1R-A2AR-D2R complex that modulates glutamate release in the presynaptic glutamate synapse. We hypothesized that the integration of changes in presynaptic glutamate release and postjunctional heteroreceptor complex signaling, where D2R plays a key role, result in no changes in the firing of the GABA anti-reward neurons, resulting in no reduction in cocaine self-administration in the present experiments.

## 1. Introduction

GPCR homoreceptor and heteroreceptor complexes in the central nervous system (CNS) are integrated through allosteric receptor–receptor interactions. These interactions modulate the recognition, signaling, and trafficking of these receptor complexes, making them important targets for drug use disorders (Fuxe et al., 2014; Navarro et al., 2015; Borroto-Escuela et al., 2017a; Lazim et al., 2021; Nguyen et al., 2021). In addition, adenosine A2AR-dopamine D2R heteroreceptor complexes have been found to play a role in cocaine reward and addiction (Borroto-Escuela et al., 2018a). Upon cocaine self-administration, modulation of antagonistic allosteric A2AR-D2RR interactions was observed in A2AR-D2R heterocomplexes located in the nucleus accumbens. Furthermore, A2AR agonists blocked cocaine-induced reinstatement of seeking cocaine (Romero-Fernandez et al., 2022).

Cocaine can also produce pathological A2AR-D2R-Sigma1R heterocomplexes that lead to a strong brake on D2R protomer signaling, causing cocaine addiction through the presence of the Sigma1R protomer (Borroto-Escuela et al., 2018a). The Sigma1R is an intracellular chaperone that can be translocated into the plasma membrane by cocaine, where it can interact with the D2R (Kourrich et al., 2012). It has been shown in previous studies that cocaine can target the Sigma1R, which can counteract its effects (Kourrich et al., 2012). However, the Sigma1R ligand OSU-6162, which in low doses (5 mg/kg) selectively binds to the Sigma1R (Sahlholm et al., 2013), failed to alter the behavior found during cocaine self-administration in rats (Borroto-Escuela et al., 2020). Nevertheless, the low dose (5 mg/kg) of OSU-6162 could still produce significant increases in the density of the A2AR-D2R and D2R-Sigma1R heterocomplexes in the nucleus accumbens shell in rats self-administering cocaine (Borroto-Escuela et al., 2020). Furthermore, the effects of the A2AR agonist CGS 21680 on its inhibition of D2R affinity, given *ex vivo*, were significantly enhanced by the OSU-6162 treatment vs. vehicle treatment. Thus, neurochemical effects on the heteroreceptor complexes were observed in the absence of changes in cocaine infusion and active lever pressing.

Based on the studies of Arvid Carlsson and Pia Steensland et al. (Lahti et al., 2007; Steensland et al., 2012; Fredriksson et al., 2019), OSU-6162 dopamine stabilizer has a direct effect on the D2R as a partial agonist in the range of 15–25 mg/kg. They hypothesized that the partial D2R agonist OSU-6162 taken in the abovementioned doses has a stabilizing function. OUS-6162 acts both on D2R located at the post-junctional level, increasing D2R signaling, and at the pre-junctional level, activating D2 auto-receptors and reducing the release of dopamine. As a result, D2R is stabilized.

In this study, we aimed to test the effect of combined threshold doses of A2AR agonist (CGS21680, 0.05 mg/kg) and Sigma1R ligand (OSU-6162, 2.5 mg/kg, to fully exclude dopamine receptor stabilizer actions) on cocaine self-administration while still producing significant changes in the A2AR-D2R heteroreceptor complexes and their allosteric receptor–receptor interactions. We anticipated that combined threshold doses of A2AR agonist and Sigma1R ligand through allosteric inhibition could counteract D2R protomer signaling. This finding would improve our understanding of heterogeneities in the ventral striatal-pallidal GABA anti-reward neurons' responses to cocaine self-administration. Additionally, this study examined the effect of cocaine self-administration with and without OSU-6162 treatment on the number of astrocytes and their process branching in the nucleus accumbens shell, considering their ability to release adenosine and ATP, which undergoes hydrolysis to adenosine extracellularly (Acton and Miles, 2017). Astrocytes can have an impact on neuronal function in the nucleus accumbens shell involving changes in their ability to alter, e.g., the allosteric interactions in A1R-A2AR heterocomplexes (Ciruela et al., 2006; Cristovao-Ferreira et al., 2013; Navarro et al., 2018) and putative A1R-A2AR-D2R heterocomplexes in the presynaptic glutamate synapses on the GABA antireward neurons modulating glutamate release and the firing of these GABA neurons.

## 2. Material and methods

### 2.1. Animals

We used male Sprague–Dawley rats (obtained from the licensed animal breeder Charles River, Sulzfeld, Germany) weighing between 250 and 270 g at the beginning of the experiment. All animals used in the study were experimentally tested. The animals were housed individually in standard plastic rodent cages in a colony room maintained at 22 ± 2°C and 55 ± 10% humidity under a 12-h light-dark cycle (lights on at 6:00 am). Rodent food (VRF1 pellets, UK) and water were available *ad libitum* except for the period of the initial lever pressing training, during which the rats were maintained on limited food. All protocols were conducted during the light phase of the light-dark cycle, between 9:00 and 15:00 h. The experiments were carried out in accordance with the European Directive 2010/63/EU and were approved by the Ethical Committee (76/2019, 2020/2019) at the Institute of Pharmacology, Polish Academy of Sciences, Krakow. The total number of subjects used for behavioral procedures was 38 (six animals did not meet the self-administration procedure criteria). For statistical analysis, a total of 32 rats were used (eight rats per group).

### 2.2. Drugs

Cocaine HCl ((1R, 2R, 3S, 5S)-3-(benzoyloxy-8-methyl-8-azabicyclo [3.2.1] octane-2-carboxylic acid methyl ester hydrochloride); Toronto Research Chemicals, Canada) was dissolved in sterile 0.9% NaCl. It was administered *i.v*. at a volume of 0.1 ml per infusion. OSU-6162 hydrochloride ((3S)-3-[3-(methyl sulfonyl)phenyl]-1-propylpiperidine hydrochloride; Tocris, UK, 2.5 mg/kg, *s.c*.) and CGS 21680 hydrochloride (4-[2-[[6-Amino-9-(N-ethyl-β-D-ribofuranuronamidosyl)-9H-purin-2-yl]amino]ethyl]benzenepropanoic acid hydrochloride; Tocris, UK, 0.05 mg/kg, *i.p*.) were dissolved in 0.9% NaCl and administered for 60 and 10 min, respectively, before the start of the 2 h self-administration session at a volume of 0.1 ml/kg.

### 2.3. Surgery

All the animals were anesthetized with ketamine HCl (Biowet, Pluawy, Poland, 75 mg/kg, *i.m*.) and xylazine (Biowet, Poland; 5 mg/kg, *i.m*.) given as a cocktail and chronically implanted with a silastic catheter in the external jugular vein, as described in a previous study (Filip et al., 2006). During the 3 days after surgery, meloxicam (*Metacam*, Boehringer Ingelheim; 5 mg/kg, *s.c*.) was used to reduce postoperative pain.

Rats were allowed 7–9 days to recover from surgery before the start of the experiments. Catheters were flushed daily with 0.2 ml of saline solution containing heparin (Biochemie, Austria; 100 U/ml) and 0.1 ml of a cephazolin solution (Biochemie GmbH, Austria; 100 mg/ml) to prevent catheter non-patency. No problems with catheter patency were reported in the tested rats.

### 2.4. Initial training (lever pressing)

During lever pressing training (2–3 days), each rat had food (VRF1 pellets, UK) limited to 25 g/day in a home cage. The animals were trained in standard operant chambers (Med-Associates, St. Albans, USA) in 2-h daily sessions. Presses on an “active” lever in the chamber resulted in the delivery of 0.1 ml of sweetened milk (SM Gostyń, Poland). After ensuring the criteria for initial training (100 rewards) under the fixed ratio (FR) as a schedule of reinforcement were met, the FR was subsequently increased to 5.

### 2.5. Cocaine self-administration

After the recovery period, all animals began lever pressing for cocaine reinforcement during daily 2-h sessions performed 6 days per week (Monday–Saturday). The house light was illuminated throughout each session. Each press on the “active” lever (FR-5 schedule of reinforcement) resulted in a 5-s infusion of cocaine (0.5 mg/kg per 0.1 ml) and a 5-s presentation of a stimulus complex (activation of the white stimulus light directly above the “active” lever and the tone generator). After each injection, there was a 20-s time-out period during which the responses were recorded but had no programmed consequences. Presses on the “inactive” lever were recorded but not reinforced. Seven days after acquisition, rats were used to complete a cocaine (0.25–0.5 mg/kg/infusion) dose-response procedure. Cocaine self-administration was conducted daily for 17 sessions. The animals exhibited a stable number of lever presses during the last three sessions, with <10% differences in their daily intake of cocaine. Following the stabilization of response rates with cocaine (0.25 mg/kg/infusion) self-administration, the animals were divided into separate groups (*n* = 8) to undergo test procedures. Vehicle, or OSU-6162, was administered daily for the three last cocaine SA sessions, while CGS 21680 was administered only before the last cocaine self-administration session. Immediately after the last cocaine self-administration sessions, animals were either euthanized (for biochemical analysis) or injected with pentobarbital and perfused intracardially (for IHC and *in situ* PLA analysis).

### 2.6. Yoked “procedure”

**Figure 1:**
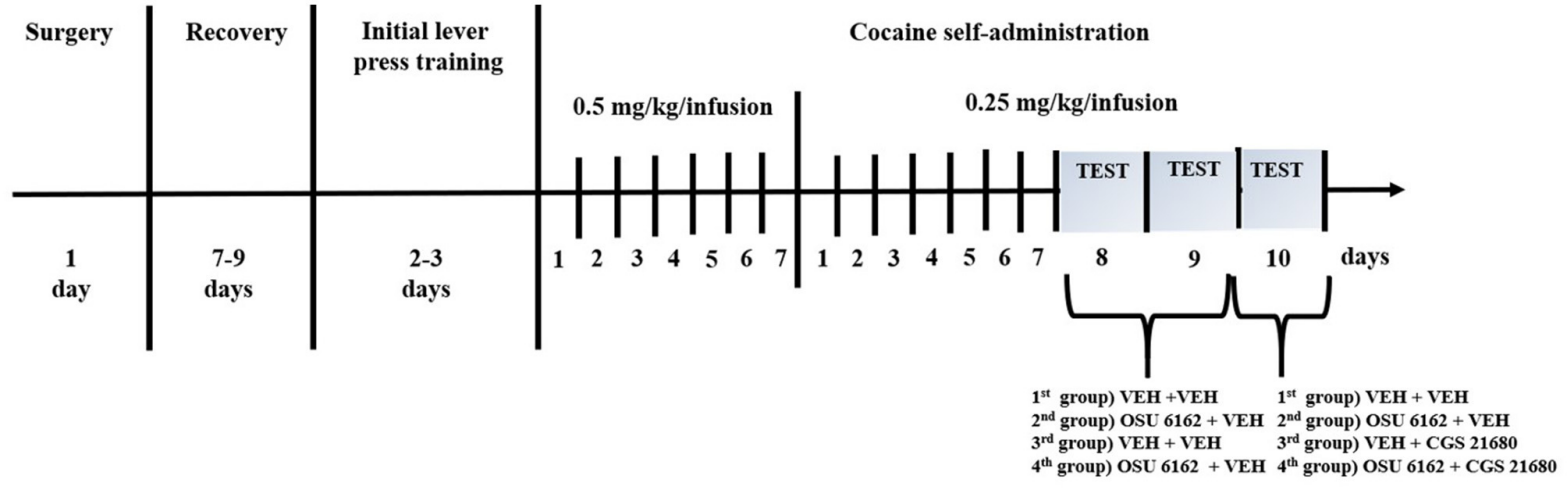
Experimental design of the study. Schematic diagram illustrating the experimental procedure. VEH, vehicle.

In yoked “procedure”, each rat that was actively self administering cocaine was assigned to the rats that passively received intravenous saline in the same amount and manner as the active animal. Lever pressing by the “yoked” rats was recorded, but it had no programmed consequences. The yoked saline group was euthanized at the same time as the corresponding group of rats that were self-administering cocaine after a 2-h experimental session of cocaine self-administration. The plan of the experiment is presented in Figure 1.

### 2.7. Biochemical binding experiments

#### 2.7.1. Membrane preparation

Frozen tissue was homogenized in an ice-cold preparation buffer using a sonicator (Soniprep 150). The buffer contained 50 mM Tris-HCl, pH 7.4, 7 mM MgCl_2_, 1 mM EDTA, a cocktail of protease inhibitors (Roche Diagnostics, Mannheim, Germany), and 0.3 IU/ml adenosine deaminase (EC 3.5.4.4, Sigma-Aldrich). The membranes were precipitated using centrifugation at 4°C for 40 min at 40,000 × *g* (Thermo Scientific, Sorvall Lynx 6000, Stockholm, Sweden) and washed through re-homogenization in the same buffer one more time. The protein concentration of the membrane homogenates was determined using the BCA Protein Assay (Pierce, Sweden), using standard bovine serum albumin. Pelleted membranes were resuspended at a concentration of 0.15 mg/ml, immediately used, or stored at −80°C until required.

#### 2.7.2. [^3^H]-raclopride competition binding experiments

[^3^H]-raclopride binding was displaced by quinpirole to determine the proportion of receptors in the high-affinity state (RH), the high-affinity value (Ki, High), and the low-affinity (Ki, Low) value. Ventral striatum membrane preparations (60 μg protein/ml) were incubated with increasing concentrations of quinpirole (0.01 nM to 1 mM) and 2 nM [^3^H]-raclopride (75 Ci/mmol, Novandi Chemistry AB, Sweden) in 250 μl of incubation buffer (50 mM Tris-HCl, 100 mM NaCl, 7 mM MgCl_2_, 1 mM EDTA, 0.05% BSA, 1 mM DTT) and 0.3 IU/ml adenosine deaminase (EC 3.5.4.4, Sigma-Aldrich). The incubation took place for 90 min at 30°C in the presence or absence of 100 nM of the A2AR agonist CGS-21680. Non-specific binding was defined by radioligand binding in the presence of 100 μM (+)-butaclamol (Sigma-Aldrich, Sweden). The incubation was terminated by rapid filtration using Whatman GF/B filters (Millipore Corp, Sweden) and a MultiScreenTM Vacuum Manifold 96-well, followed by five washes (250 μl per wash) with ice-cold washing buffer (50 mM Tris-HCl pH 7.4). The filters were dried, 5 ml of scintillation cocktail was added, and the amount of bound ligand was determined after 12 h by liquid scintillation spectrometry.

### 2.8. *In situ* proximity ligation assay (*in situ* PLA)

To study the effects of OSU-6162, a Sigma1R ligand, in low doses on the D2R-A2AR and D2R-Sigma1R heteroreceptor complexes and the density changes after cocaine self-administration, *in situ* PLA was performed as described previously (Borroto-Escuela et al., 2017b). Formalin-fixed, free-floating brain sections (30 μm-thick, cut using a cryostat) at the Bregma level (1.0 mm) from rats after cocaine self-administration were employed using the following primary antibodies: rabbit monoclonal anti-A2AR (AB1559F, 1:250; Millipore, Sweden), mouse monoclonal anti-D2R (MABN53, 1:600, Millipore, Sweden), and rabbit monoclonal antisigma1R (ab53852, 1:500, Abcam, Sweden). The primary antibodies had been validated previously by means of immunohistochemistry in both rat brain tissue and the HEK293 cell line (Borroto-Escuela et al., 2017b, 2018b; Feltmann et al., 2018). To localize the heteroreceptor complexes in relation to neurons and astrocytes in the brains, traditional immunohistochemistry (IHC) was employed in parallel to *in situ* PLA. For this purpose, a glial marker (GFAP) and a nucleic marker (DAPI) were used. Control experiments for *in situ* PLA procedures were performed in free-floating, formalin-fixed rat brain sections employing only one primary antibody (mouse monoclonal anti-D2R (MABN53), 1:600, Millipore, Sweden). The *in situ* PLA analysis of this negative control showed 15.6% false-positive clusters compared to the positive control group value (100%). This false-positive signal was reduced even further (<4%) when the brain sections were kept in a citric acid buffer for 45–60 min at 65°C prior to the primary antibody incubation. Control experiments with similar results were also performed in cells transfected with cDNAs encoding only one type of receptor. The PLA signal was visualized and quantified using a Leica TCS-SL SP5 confocal microscope (Leica, USA) and the Duo Link Image Tool software. Briefly, fixed free-floating rat brain sections (stored at −20°C in Hoffman solution) were washed four times with PBS and quenched with 10 mM glycine buffer for 20 min at room temperature. Then, after three PBS washes, incubation took place with a permeabilization buffer [10% fetal bovine serum (FBS) and 0.5% Triton X-100 or Tween 20 in Tris buffer saline (TBS), pH 7.4] for 30 min at room temperature. The sections were washed twice for 5 min each with PBS at room temperature and incubated with the blocking buffer (0.2% BSA in PBS) for 30 min at room temperature. The brain sections were then incubated with the primary antibodies diluted to a suitable concentration in the blocking solution for 1–2 h at 37°C or at 4°C overnight. The sections were washed two times the day after, and the proximity probe mixture (minus and plus probes; for details, please refer to the Duolink instructions) was applied to the sample and incubated for 1 h at 37°C in a humidity chamber.

The unbound proximity probes were removed by washing the slides twice for 5 min each time with the blocking solution kept at room temperature under gentle agitation. The sections were incubated with the hybridization-ligation solution [BSA (250 g/ml), T4 DNA ligase (final concentration of 0.05 U/μl), Tween-20 (0.05%), NaCl 250 mM, ATP 1 mM, and the circularization or connector oligonucleotides (125–250 nM)] and incubated in a humidity chamber at 37°C for 30 min. The excess of connector oligonucleotides was removed by washing twice for 5 min each with the washing buffer A [Sigma-Aldrich, Duolink Buffer A (8.8 g NaCl, 1.2 g Tris Base, 0.5 ml Tween 20)] dissolving in 800 ml of high-purity water (pH 7.4) at room temperature under gentle agitation, and the rolling circle amplification buffer was added to the sections and incubated in a humidity chamber for 100 min at 37°C. Then, the sections were incubated with the detection solution through hybridization (fluorescent oligonucleotide probes) in a humidity chamber at 37°C for 30 min. In the last step, the sections were washed twice in the dark for 10 min each with the washing buffer B (Sigma-Aldrich, Duolink Buffer B (5.84 g NaCl, 4.24 g Tris Base, 26.0 g Tris-HCl), dissolved in 500 ml of high-purity water, pH 7.5) at room temperature under gentle. The free-floating sections were put on a microscope slide, and a drop of appropriate mounting medium containing DAPI, giving blue staining of the nuclei (e.g., VectaShield or Dako), was applied. The coverslip was placed on the section and sealed with nail polish. The sections were protected against light and stored for several days at −20°C before confocal microscope analysis.

### 2.9. GFAP IHC and *in situ* PLA image acquisition and analysis

Images of the samples were taken with a confocal microscope, i.e., the LEICA TCS-SL SP5 confocal microscope. Three different areas of the nucleus accumbens shell (AcbSh) were selected, from which two randomly chosen magnified sample fields (150 x 150μm) were used for image acquisition, yielding a total of six pictures per animal. Images were inspected before being analyzed to exclude unrepresentable pictures, e.g., pictures containing blood vessels (which naturally attract astrocytes). As described previously, nuclei and PLA signal quantification were performed with DuoLink Image Tool software (Borroto-Escuela et al., 2013, 2016). Astrocyte quantification, including branch analysis, was performed with the open-source Fiji ImageJ software using the Ridge detection plugin. Using Sholl morphological analysis, we evaluated the development of astrocytes and quantified their morphological and structural changes in cocaine-self-administering rats vs. yoked saline groups. Sholl analysis was performed by overlaying circles of increasing diameter (in 1.5 mm steps) out from the center of the astrocyte soma and by counting the number of intersections the astrocytes make with each circle. A total of 10 astrocytes per group were investigated.

### 2.10. Statistical analysis

Data were analyzed using GraphPad Prism 5.0 (GraphPad Software Inc., San Diego, CA). All the data were shown as means ± SEM. In behavioral experiments, the number of responses on the “active” and “inactive” levers or the number of cocaine infusions were analyzed using the factorial analysis of variance (ANOVA) for factors (groups and levers). Data from competition experiments were analyzed by nonlinear regression analysis. The absolute values and the percent changes induced by A2AR agonist CGS 21680 in the dopamine D2R high-affinity, low-affinity, and proportion of receptors in the high-affinity state were evaluated using a paired Student's *t*-test and a non-parametric Mann–Whitney *U-*test, respectively. Data from *in situ* PLA experiments showing cluster density (clusters per nucleus per sampled field) were analyzed using a one-way ANOVA followed by a *post-hoc* Tukey's test. The number of rats (*n*) in each experimental condition is indicated in the figure legends. Data from GFAP experiments were analyzed using the Mann–Whitney *U*-test and a two-way ANOVA (see Supplementary Figure 1). A *p-*value of 0.05 and lower was considered statistically significant.

## 3. Results

### 3.1. Behavioral, pharmacological analysis of cocaine self-administration

**Figure 2:**
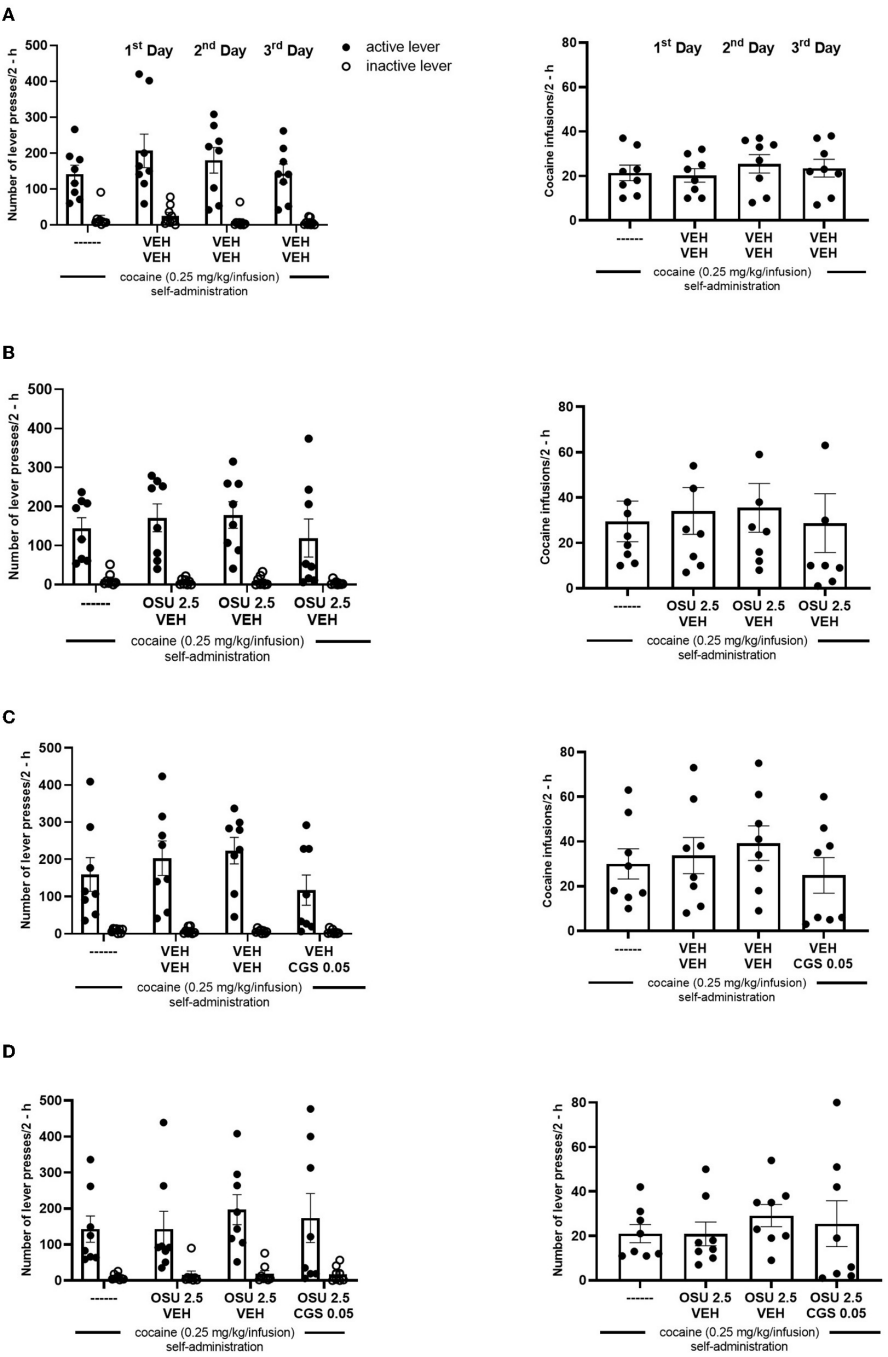
**(A)** Effects of repeated administration of vehicle (VEH; 0.9% NaCl, *s.c. or i.p*.), **(B)** OSU-6162 (OSU; 2.5 mg/kg, *s.c*.), **(C)** CGS 21680 (CGS; 0.05 mg/kg, *i.p*.), and **(D)** OSU-6162 (OSU; 2.5 mg/kg, *s.c*.) plus CGS 21680 (CGS; 0.05 mg/kg, *i.p*.) treatments on cocaine (0.25 mg/kg/infusion) self-administration under the FR-5 schedule of reinforcement in rats. The numbers of “active” and “inactive” lever presses (left panels) as well as cocaine infusions (right panels) after 2-h sessions are expressed as the means ± SEM of the data from eight rats/group.

After 17 sessions, the rats completed cocaine self-administration (i.e., they received ≥23 infusions at 2-h sessions under 0.25 mg/kg/infusion of cocaine) and displayed <10% variation in the number of cocaine infusions (Figure 1). The mean group number of cocaine infusions per day during the last three self-administration days varied from 23 to 28. The total cocaine intake during the 17 days (means ± SEM of 8 rats) was 103 ± 10 mg/rat for the vehicle group, 148 ± 33 mg/rat for the OSU-6162 group, 123 ± 18 mg/rat for the CGS 21680 group (see Figure 2 for more details), and 134 ± 15 mg/rat for the OSU-6162 + CGS 21680 groups (Figure 2).

**Figure 3:**
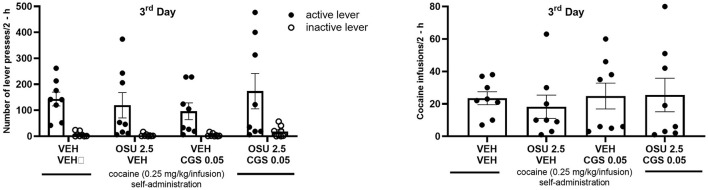
Effects of repeated administration of OSU-6162 (OSU; 2.5 mg/kg, *s.c*.) with or without combination with CGS 21680 (CGS; 0.05 mg/kg, *i.p*.) on cocaine (0.25 mg/kg/infusion) self-administration under the FR-5 schedule of reinforcement in rats. The numbers of “active” and “inactive” lever presses (left panels) as well as cocaine infusions (right panels) after 2-h sessions are expressed as the means ± SEM of the data from eight rats/group.

No difference was observed between the groups assigned as vehicles or the OSU-6162 or CGS 21680 groups in the context of “active” and “inactive” lever presses and cocaine infusions (Figure 2). OSU-6162 in a dose of 2.5 mg/kg given in combination with CGS 21680, 0.05 mg/kg, did not change the number of “active” and “inactive” lever presses [group × lever *F*_(3,56)_ = 0.174, *p* = 0.91; group *F*_(3,56)_ = 0.045, *p* = 0.71; lever *F*_(1,56)_ = 28.58; *p* = 0.0001] and cocaine infusions [*F*_(3,28)_ =0.14; *p* = 0.09; Figure 3].

### 3.2. Studies with *in situ* PLA on the effects of very low doses of OSU-6162 and CGS 21680 in cocaine self-administration on A2AR-D2R heteroreceptor complexes of the nucleus accumbens shell

**Figure 4:**
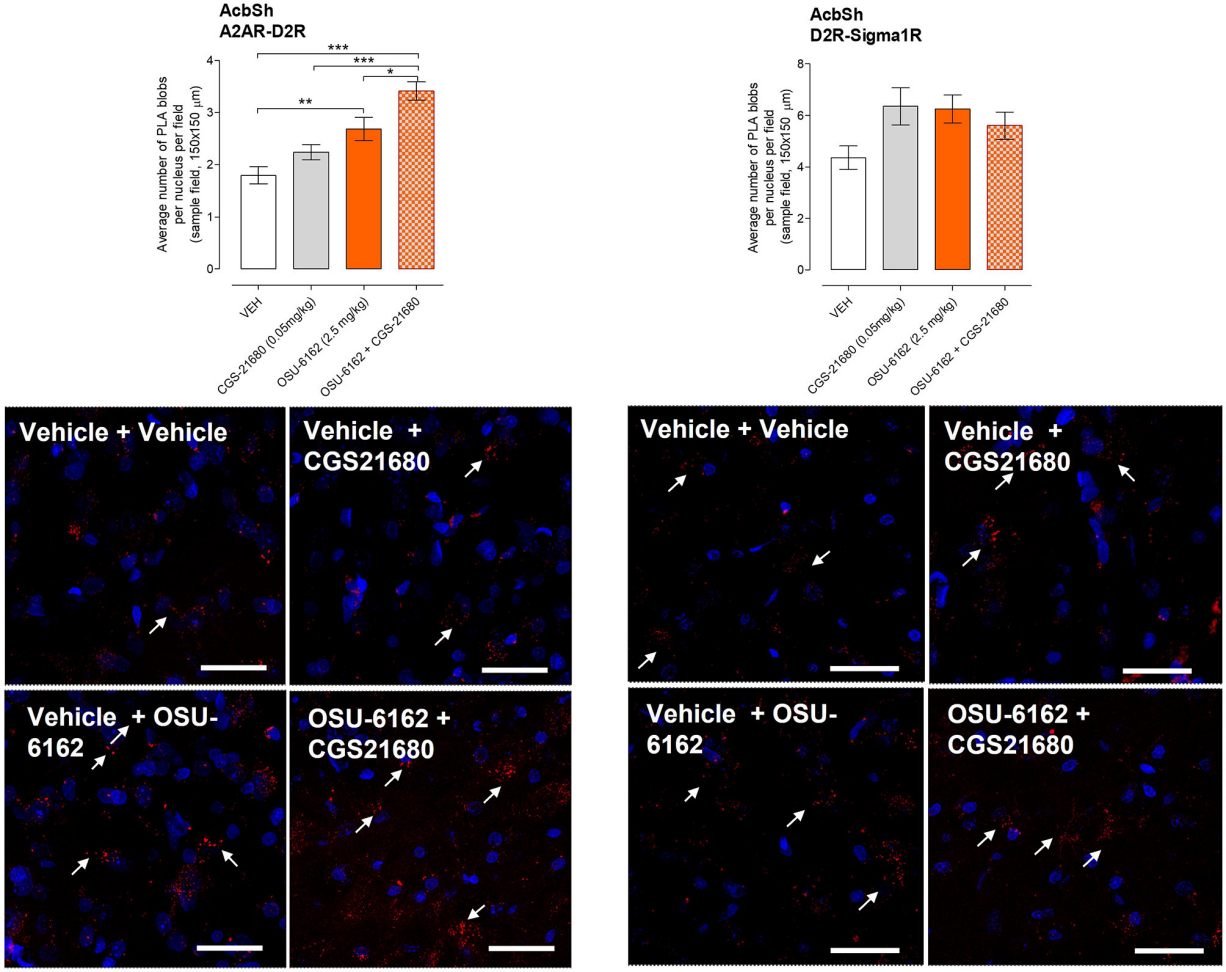
Effects of pharmacological treatment on cocaine self-administration (SA) on A2AR-D2R and D2R-Sigma1R heteroreceptor complexes using proximity ligation assay (PLA). (Bottom panels) The heteroreceptor complexes are shown as red puncta, and their average number has been determined per nucleus per sample field using confocal laser microscopy in the nucleus accumbens shell (AcbSh). (Top panels) The effects of CGS 21680 (CGS; 0.05 mg/kg, *i.p*.) and/or OSU-6162 (OSU; 2.5 mg/kg, *s.c*.) in cocaine-self-administrating rats using the same doses as reported in Figure 5 are shown in terms of density changes in their heterodimers compared to the density found in vehicle (VEH; 0.9% NaCl) treated cocaine-self-administrating rats. Significant changes were observed in the A2AR-D2R heterocomplexes as studied in the nucleus accumbens shell. The OSU-6162 treatment cocaine SA group was significantly increased compared to the vehicle SA group (**p < 0.01). The combined treatment group showed the strongest increase in its density with highly significant increases against the vehicle and CGS 21680 SA groups (***p < 0.001) and with a significant increase against the OSU-6162 group (*p < 0.05). Statistical analysis was performed by a one-way ANOVA followed by the Tukey *post hoc* analysis to obtain the means ± SEM of the data from four rats/group.

OSU-6162 treatment in cocaine-self-administrating rats at the very low dose of 2.5 mg/kg, given once daily over 3 days, produced a significant increase in the density of red fluorescent PLA puncta (A2AR-D2R heteroreceptor complexes) per nucleus in the sampled field in the nucleus accumbens shell vs. vehicle-treated cocaine-self-administrating rats (Figure 4). A combination with a low dose (0.05 mg/kg) of the A2AR agonist CGS 21680, which by itself did not change the density of the PLA complexes, significantly enhanced the density of the red PLA complexes in the nucleus accumbens shell compared to treatment with OSU-6162 or CGS 21680 alone and especially with the vehicle-alone treatment (Figure 4). Representative images of the A2AR-D2R density increases in red positive puncta in the nucleus accumbens shell are shown upon combined treatment with OSU-6162 and CGS 21680 compared to vehicle treatment (Figure 4). For comparison, the D2R-Sigma1R heteroreceptor complexes were studied in cocaine self-administration after single or combined treatment with a low dose (0.05 mg/kg) of the A2AR agonist CGS 21680 and a very low dose of 2.5 mg/kg of Sigma1R ligand OSU-6162. Daily treatment for 3 days did not produce a significant increase in the density of these heteroreceptor complexes in the nucleus accumbens shell vs. the vehicle-treated group in cocaine self-administration (Figure 4). An overall high density of A2AR-D2R and D2R-Sigma1R PLA positive clusters was found in the nucleus accumbens of the ventral striatum (Figure 4, bottom panels) based on the average number of clusters per nucleus (in blue) per sample field. They were both significantly different from the number of PLA positive clusters in the myelinated bundles of the crus cerebri (CC) and the anterior limb of the anterior commissure (aca), and negative controls were regarded as background values (please refer to Supplementary Figure 2).

### 3.3. Effects of single and combined systemic OSU-6162 and CGS 21680 treatment on the antagonistic allosteric A2AR-D2R receptor-receptor interactions in the ventral striatum in cocaine self-administering rats

Co-treatment was performed as mentioned above in the PLA experiments with OSU-6162 (2.5 mg/kg) and CGS 21680 (0.05 mg/kg). A2AR agonist (CGS 21680) was given on the last day 50 min after OSU-6162 with its 60-min pretreatment before the 120-min long cocaine self-administration session prior to euthanasia. The combined treatment resulted in a marked and significant increase in D2R K_*i, High*_, and D2R.

**Figure 5:**
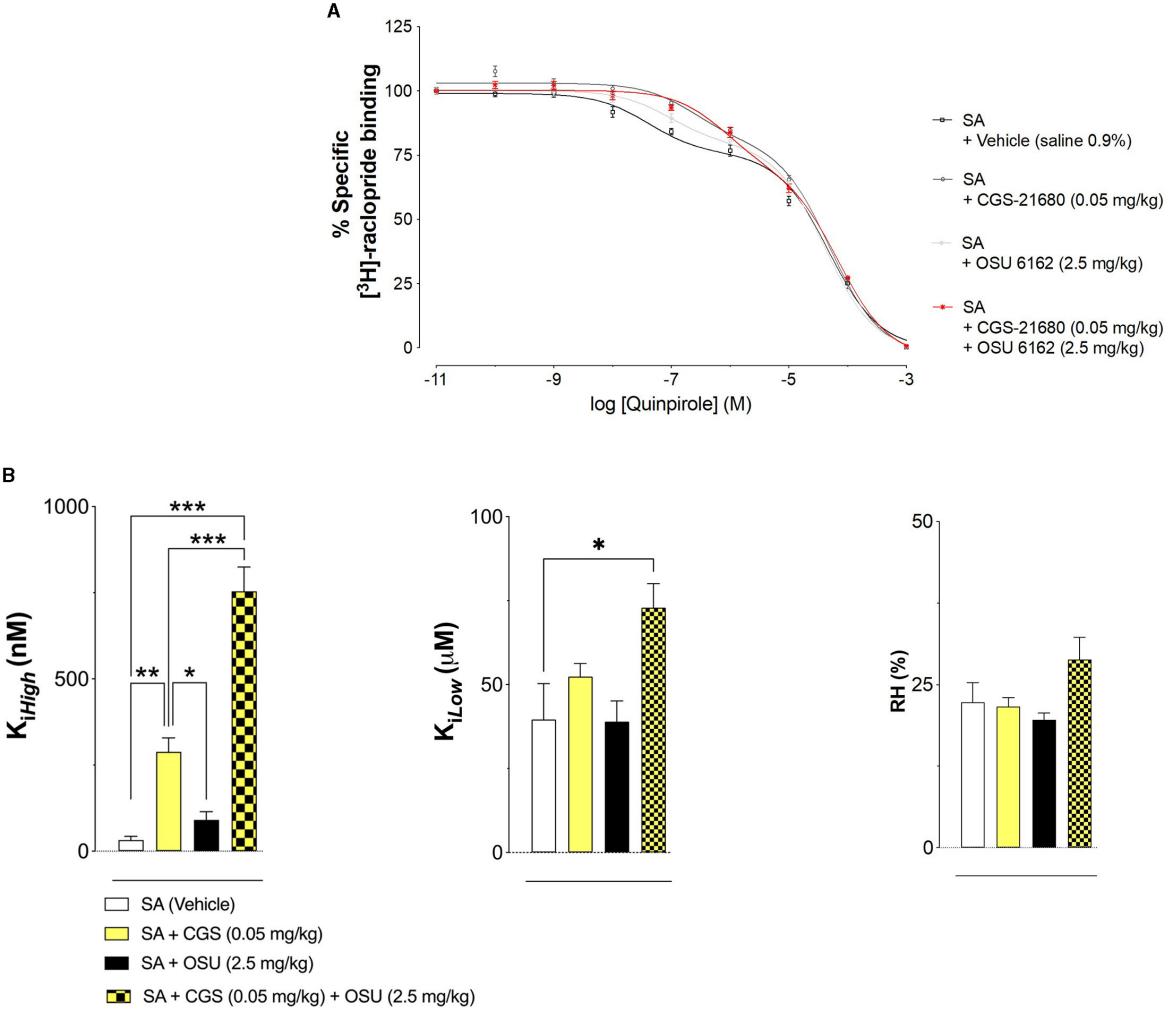
**(A)** Effects of pharmacological treatments on cocaine self-administration (SA) on D2R affinity. [^3^H]-raclopride/quinpirole competition binding experiments were performed to study changes in D2R affinities produced by A2AR agonist CGS 21680 (CGS 0.05 mg/kg) and/or the Sigma 1R ligand OSU-6162 (OSU 2.5 mg/kg) and compared with vehicle (control group; VEH: 0.09% NaCl). Competition experiments involved the D2-likeR antagonist [^3^H]-raclopride binding vs. increasing concentrations of quinpirole in ventral striatal membrane preparations from the control group (60 μg/ml). Non-specific binding was defined as the binding in the presence of 100 μM (+) –butaclamol. [^3^H]-raclopride/quinpirole competition curves were obtained based on the values of four rats with each experiment performed in duplicate. The binding values were given in percentage of specific binding at the lowest concentration of quinpirole employed **(B)**. Modulation of D2R affinity was found with CGS 21680 and OSU-6162 and their combined treatment which induced changes in the high affinity value (D2R K_i__high_). In the low-affinity component (K_ilow_), a significant change (*p < 0.05) was only found in the combined treatment group with no significant changes in the values of the proportion of the D2R-like receptors in the high-affinity state (RH; upper right and lower left). Means ± SEM are given from four independent experiments performed in duplicate. Statistical analysis was performed by a one-way ANOVA followed by the *post hoc* analysis, **p < 0.01. The group of rats exposed to combined treatment was significantly different compared to the group receiving only vehicle treatment.

Figure 5 shows the effects of pharmacological treatments on cocaine self-administration (SA) on D2R high-and low-affinity K_i_ values in the ventral striatum produced by A2AR agonist CGS 21680 (CGS 0.05 mg/kg) and/or the Sigma 1R ligand OSU-6162 (OSU 2.5 mg/kg) and compared with vehicle (control group; VEH: 0.09% NaCl). It shows a strong reduction of D2R affinity in the nucleus accumbens after combined treatment with OSU-6162 and CGS 21680, involving both high-and low-affinity K_i_ values.

Treatment with either OSU-6162 or CGS 21680 alone did not lead to any changes in these D2R K_i_ values.

### 3.4. Effects of cocaine self-administration vs. yoked procedure on astrocytes in the nucleus accumbens shell

#### 3.4.1. Density of astrocytes

**Figure 6:**
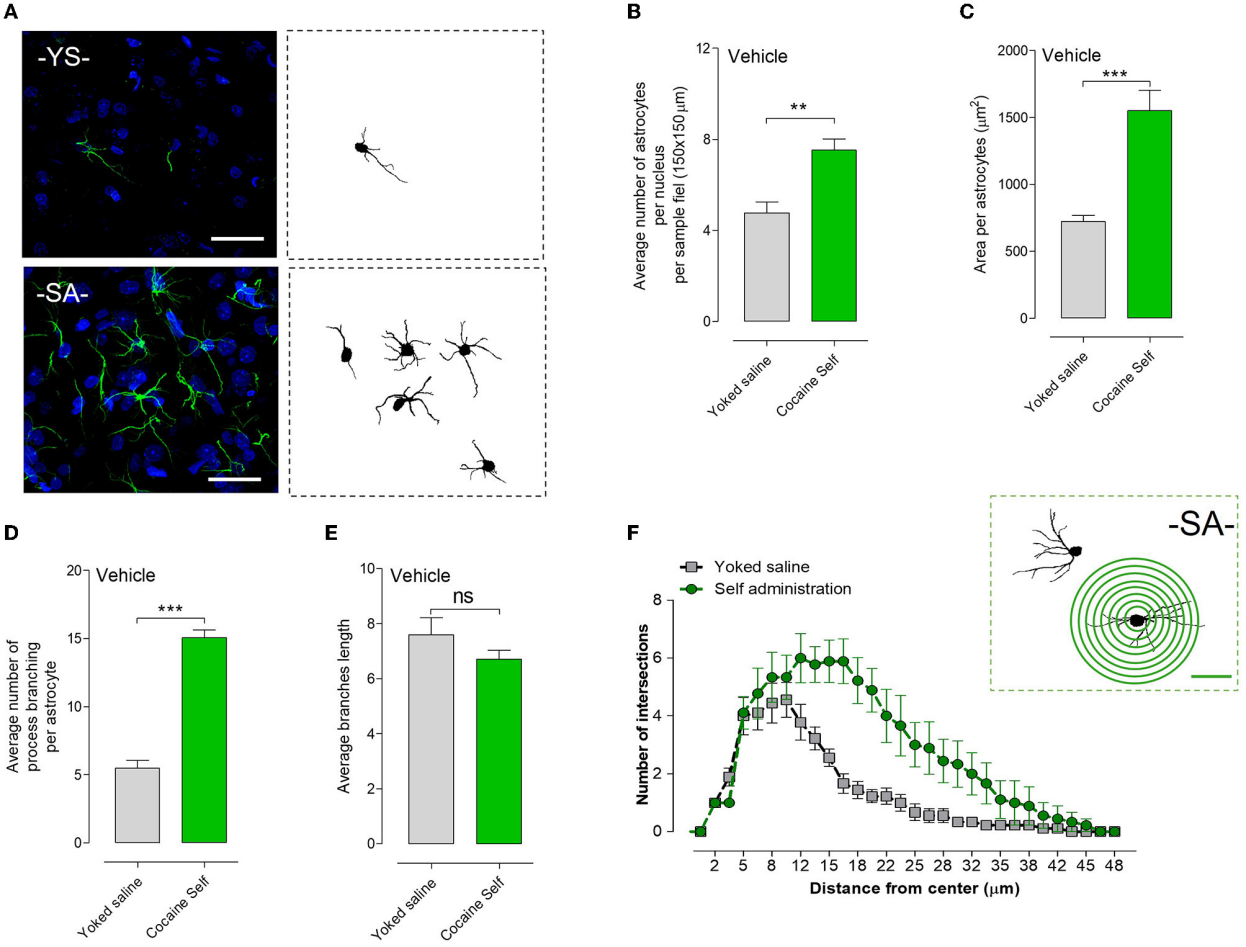
Structural changes in astrocytes after cocaine self-administration compared to the yoked saline controls procedure in rats in terms of the number of astrocytes and their process branching in the nucleus accumbens shell. **(A)** Representative GFAP images of the GFAP-positive astrocytes with their process branching are presented after cocaine self-administration or after the yoked saline procedure are also given. **(B)** Cocaine self-administration vs. yoked saline controls produced a significant increase in the density of astrocytes per nucleus per sample field (**p < 0.01, Mann–Whitney *U*-test, means ± SEM, *n* = 8 rats) in the nucleus accumbens shell, visualized in green with GFAP immunoreactivity. **(C)** In addition, a significant change in the area was observed. **(D)** Cocaine self-administration caused a highly significant and substantial increase in the number of GFAP-positive branches per astrocyte (***p < 0.001, Mann–Whitney test, means ± SEM, *n* = 8 rats) in the nucleus accumbens shell vs. yoked saline controls. **(E)** However, a non-significant (ns) change in the average branch length was observed. **(F)** illustrates the Sholl method for an astrocyte, labeled GFAP, in cocaine self-administration of an accumbens shell sample. A total of 10 astrocytes per group were investigated, and the results clearly indicated an increase in the number of intersections (hence cell processes) with the entire range of astrocytes being compared between the cocaine-self administration group and the yoked saline group in **(B, C)**.

Cocaine self-administration vs. yoked saline controls produced a significant increase in the density of astrocytes per nucleus per sample field (^**^p < 0.01, Mann–Whitney *U*-test, mean, and SEM) in the nucleus accumbens shell, visualized in green with GFAP immunoreactivity (Figures 6A, B). In addition, a significant change in the area was observed (Figure 6C).

#### 3.4.2. Astrocytic process branching

Cocaine self-administration caused a highly significant and substantial increase in the number of GFA-positive branches per astrocyte (^***^p < 0.001, Mann–Whitney *U*-test, mean, and SEM) in the nucleus accumbens shell vs. yoked saline controls (Figure 6D). Representative GFAP images of the GFAP-positive astrocytes with their process branching are presented after cocaine self-administration or yoked procedures (Figure 6A). However, a non-significant change in the average branch length between the cocaine self-administration and the yoked saline groups was observed (Figure 6E).

#### 3.4.3. Sholl morphological analysis of astrocytes

Using Sholl morphological analysis, we evaluated the development of astrocytes and quantified their morphological and structural changes after cocaine self-administration vs. yoked delivery of saline. Figure 6F (Top right) illustrates the Sholl method for an astrocyte labeled for GFAP in a cocaine self-administration accumbens shell sample. A total of 10 astrocytes per group were investigated, and the results indicated an increase in the number of intersections across the entire range of distance in the cocaine-self-administration group vs. the yoked saline group (Figure 6F).

### 3.5. Effects of OSU-6162 (2.5 mg/kg) on astrocyte morphology in cocaine self-administration model and yoke saline rats

**Figure 7:**
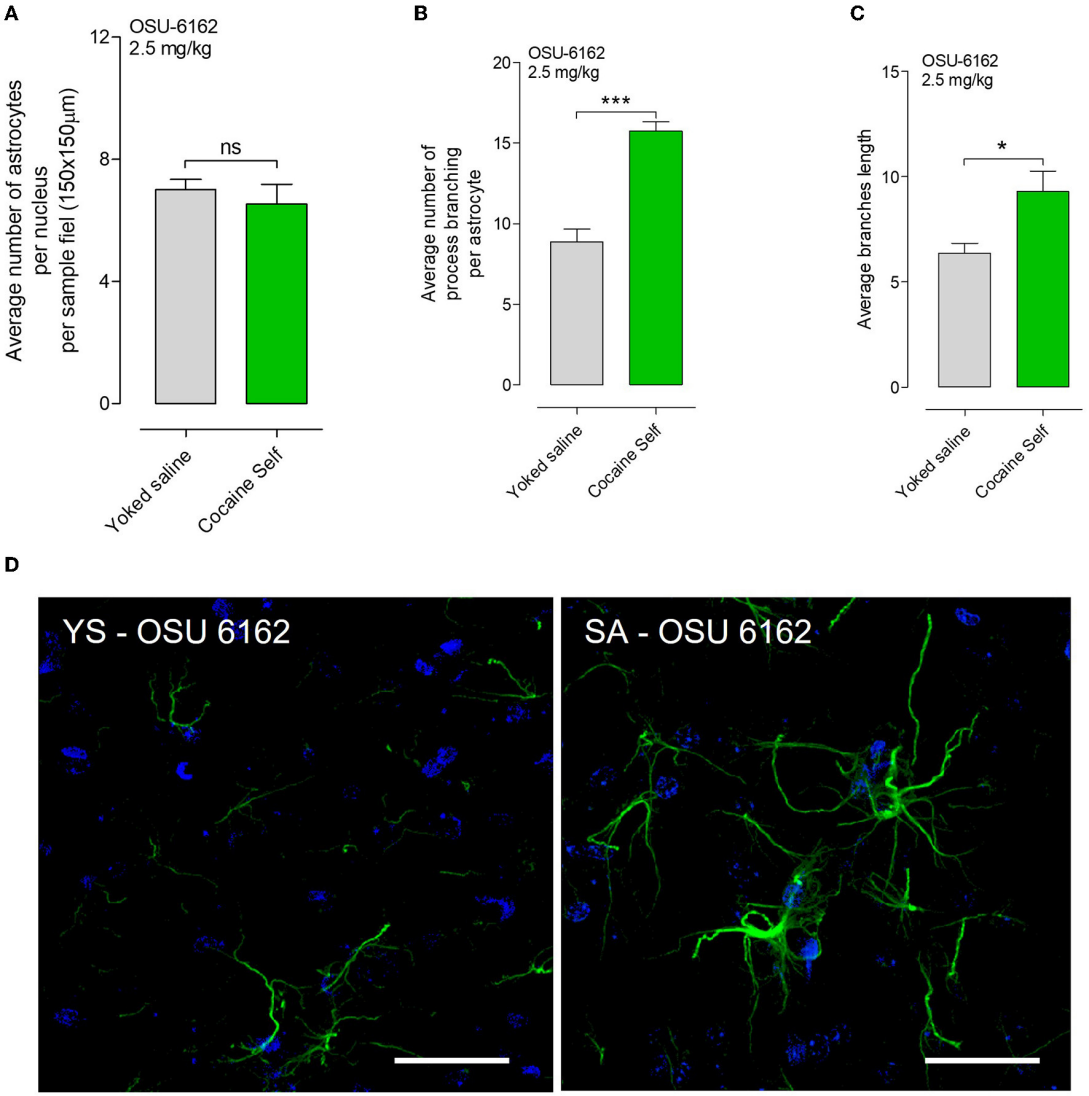
**(A)** OSU-6162 with low dose (OSU 2.5 mg/kg) treatment produced a non-significant (ns) change in the density of astrocytes per nucleus per sample field (*p* > 0.05, Mann–Whitney *U*-test, means ± SEM, *n* = 8 rats) in the nucleus accumbens shell, visualized in green with GFAP immunoreactivity after cocaine self-administration vs. yoked saline controls. **(B)** However, OSU-6162 treatment caused a highly significant and substantial increase in the number of GFAP-positive branches per astrocyte (***p < 0.001, Mann–Whitney *U*-test, means ± SEM, *n* = 8 rats) in the nucleus accumbens shell of cocaine-self administration rats vs. yoked saline controls. **(C)** Moreover, significant changes in the average branch length were observed (*p < 0.01, Mann–Whitney *U*-test, means ± SEM, *n* = 8 rats). **(D)** Representative GFAP images of the GFAP-positive astrocytes with their process branching are presented in cocaine self-administration or yoked procedure after OUS-6162 low-dose treatment (OSU 2.5 mg/kg). There is a clear-cut increase visualized in the GFAP branching of the astrocytes after OSU6162 treatment in cocaine self-administration compared to its treatment in yoked saline rats.

No significant difference was found in the density of astrocytes per nucleus per sample field (*p* > 0.05, Mann–Whitney test, mean, and SEM), visualized in green with GFAP immunoreactivity, in rats treated with OSU-6162 before cocaine self-administration compared to the yoked saline control group in the nucleus accumbens shell (Figure 7A). However, OSU-6162 2.5 mg/kg alone caused a highly significant increase in the number of GFAP-positive branches per astrocyte (^***^p < 0.001, Mann–Whitney *U*-test, mean, and SEM) in the nucleus accumbens shell vs. yoked saline controls (Figure 7B). In addition, a significant change in the average branch length after OSU-6162 treatment was observed in the cocaine self-administration group vs. the yoked saline group (Figure 7C). Representative GFAP images of the GFAP-positive astrocytes with their process branching are presented after OSU-6162 treatment in cocaine self-administration or yoked procedures (Figure 7D).

We also performed a two-way ANOVA analysis to test the effects of treatment (vehicle vs. OSU-6162) and drug use (cocaine vs. yoked saline) on the average number of astrocytes, average number of branches per astrocyte, and branch length (see Supplementary Figure 1). The analysis showed significant interactions between the two factors for all three measures (*p* < 0.01 for an average number of astrocytes and branch length and *p* < 0.05 for the average number of branches per astrocyte). Furthermore, we found that the effect of OSU-6162 was distinctly different in the yoked saline group and the cocaine self-administration group. Specifically, in the yoked saline group, treatment with OSU-6162 produced a significant increase in the mean number of astrocytes and in the number of branches per astrocyte while resulting in no changes in the average branch length. In contrast, in the cocaine self-administration group, the mean number of astrocytes and the number of branches per astrocyte were not altered by the low-dose treatment with OSU-6162, while a significant increase in the average branch length was observed. These findings suggest that the effects of OSU-6162 on astrocytes are dependent on both drug use and treatment type.

## 4. Discussion

Previous studies have failed to demonstrate that the monoamine stabilizer OSU-6162 (Steensland et al., 2012) in a low dose (5 mg/kg), which mainly activates the Sigma1R (Sahlholm et al., 2013), altered cocaine self-administration in terms of active lever pressing and total cocaine intake (Borroto-Escuela et al., 2020). However, experiments involving 3-day treatments with OSU-6162 have revealed significant increases in the density of the A2AR-D2R and D2R-Sigma1R heterocomplexes in the nucleus accumbens shell during cocaine self-administration. This may also involve an increased formation of putative trimeric A2AR-D2R-Sigma1R heterocomplexes (Pinton et al., 2015a,b; Borroto-Escuela et al., 2018a). When an A2AR agonist was added *ex vivo* to membrane preparations from OSU-6162-treated rats undergoing cocaine self-administration, a substantial and significant enhancement of the A2AR-mediated allosteric inhibition of the D2R protomer affinity was observed (Borroto-Escuela et al., 2020). The mechanism may involve a cocaine-induced increase in the expression of Sigma1R in the ventral striatum (Romieu et al., 2002; Pinton et al., 2015a,b; Borroto-Escuela et al., 2020).

The present experiments further explored the previous study on the pharmacology of cocaine self-administration by administering an even lower dose of OSU-6162 (2.5 mg/kg) given daily for 3 days, along with systemic treatment with a threshold dose of the A2AR agonist CGS 21680 (0.05 mg/kg), with or without combined systemic treatment with OSU-6162. No significant effects of these treatments on cocaine self-administration were observed, including active lever pressing and total cocaine intake. However, it was possible to demonstrate that combined treatment with a threshold dose of OSU-6162 and CGS 21680 caused significant increases in the A2AR-D2R heterocomplexes in the nucleus accumbens shell compared to vehicle-treated animals, while there was no significant change in the densities of the D2R-Sigma1R heteroreceptor complexes. Treatment with a low dose of OSU-6162 alone but not with CGS 21680 alone produced significant but reduced increases in the density of A2AR-D2R heteroreceptor complexes compared to vehicle-treated animals in the nucleus accumbens shell.

These results enhanced the impact of previous studies. Thus, it is important to understand why there were no reductions in cocaine self-administration despite evidence that the A2AR agonist and Sigma1R ligand, when combined, significantly increased the densities of the A2AR-D2R heterocomplexes in the nucleus accumbens shell. Moreover, enhanced antagonistic A2A-D2R interactions developed upon combined treatment with the Sigma1R ligand in these heterocomplexes, reducing D2R function, which should bring down cocaine self-administration. Based on the neurochemical results, it seems likely that a significant molecular target can be the putative A2AR-D2R-Sigma1R heterocomplex, which was located extrasynaptically in the postsynaptic part of the glutamate synapses on the ventral striatal-pallidal GABA antireward neurons (Borroto-Escuela et al., 2018a; Wydra et al., 2020). However, another mechanism involved in reducing the effects of the inhibition of the D2R protomer signaling in the A2AR-D2R-Sigma1R heterocomplex on cocaine self-administration can be a substantial increase in the number of astrocytes per sampled field and of branches per astrocyte in the nucleus accumbens shell compared to the yoked saline animals.

The cocaine self-administration-induced changes in the mean number of astrocytes and the number of branches per astrocyte were not altered by the low-dose treatment with OSU-6162. However, OSU-6162 treatment in the yoked saline group significantly increased the mean number of astrocytes and the number of branches per astrocyte. The latter results indicate that Sigma 1R can be involved in mediating certain structural changes in astrocytes in the absence of cocaine.

The increased structural plasticity developed in astrocytes upon cocaine self-administration supported the possibility that astrocytes may modulate cocaine actions on neuronal function in the nucleus accumbens shell. It may develop via the alteration of astrocytic-neuronal crosstalk through changes in the release of astrocytic signals, especially adenosine and ATP, with hydrolysis to adenosine (Acton and Miles, 2017). They operate via astrocytic volume transmission (Borroto-Escuela et al., 2015; Fuxe et al., 2015; Fuxe and Borroto-Escuela, 2016) to reach and activate, especially, neuronal A1R protomers with a higher affinity for adenosine than A2AR (Canals et al., 2005; Ciruela et al., 2006; Cristovao-Ferreira et al., 2013). They may form presynaptic A1R-A2AR heterocomplexes (Ciruela et al., 2006; Cristovao-Ferreira et al., 2013) that can inhibit the A2AR protomer signaling in the cortical-striatal glutamate terminals, forming synapses on the ventral striatal-pallidal GABA anti-reward neurons. It has also been proposed that A1R-A2AR-D2R higher-order complexes may exist on the presynaptic part of the glutamate synapses on the GABA anti-reward neurons in the nucleus accumbens.

Consequently, the reduced inhibition of presynaptic D2R protomer signaling by the A2AR protomer through its inhibition by the A1R protomer may lead to increased inhibition of glutamate release by the presynaptic D2R protomer onto the ventral striatal-pallidal GABA antireward neurons. Further, the astrocytic release of adenosine may contribute to the reduction of activity in the GABA anti-reward neurons due to the reduction of their glutamate drive through preferential A1R protomer activation, inhibiting the A2AR protomer, leading to reduced inhibition of the D2R protomer signaling, which causes enhanced D2R mediated inhibition of glutamate release from the presynaptic region of the glutamate synapse. Such events should aim to reduce the activity of the GABA anti-reward neurons through the reduction of their glutamate drive, which favors the enhancement of cocaine self-administration.

As stated above, a possible explanation for the lack of inhibition of cocaine self-administration in the current experiments may be the cocaine-induced increase in the number of astrocytes and their branching process in the nucleus accumbens shell. This increase can lead to increased extracellular levels of adenosine that are released from astrocytes (Acton and Miles, 2017). Adenosine has an enhanced affinity for the A1R vs. the A2AR and may, therefore, preferentially activate the A1R protomer upon cocaine self-administration, which inhibits the A2AR protomer signaling in A1R-A2AR heterodimers (Ciruela et al., 2006). In the glutamate synapses on the GABA anti-reward neurons, presynaptic A1R-A2AR-D2R heterocomplexes are proposed to exist, with allosteric inhibition of the A2AR protomer being set free from the presynaptic D2R protomer signaling to inhibit glutamate release from the glutamate synapse onto the GABA anti-reward neurons, reducing their firing of the GABA anti-reward neurons. The combined balance of reduced glutamate release onto the GABA antireward neurons and reduced D2R protomer inhibitory signaling in the A2AR-D2R-Sigma1R heterocomplexes located close to the glutamate synapses on these GABA anti-reward neurons may be the mechanism for the lack of change in cocaine self-administration in the current experiments. The post-junctional D2R protomer-mediated inhibition of the GABA anti-reward neurons is lost through presynaptic D2R protomer-mediated inhibition of glutamate release, resulting in a lack of changes in cocaine self-administration.

Taken together, the present findings help to understand the mechanism that led to the failure of combined A2AR agonist and Sigma 1R ligand treatment with their subthreshold dose on cocaine self-administration despite significant and substantial increases in the density of the A2AR-D2R heterocomplexes in the nucleus accumbens. The explanation may be an increased release by cocaine of ATP and adenosine from astrocytes in the nucleus accumbens shell and a putative integration of presynaptic glutamate release and post-junctional heteroreceptor complex signaling where D2R plays a key role. The potential existence of such integrative mechanisms between presynaptic and postjunctional mechanisms in glutamate synapses on GABA antireward neurons is highly interesting and should be further investigated.

## Data availability statement

The raw data supporting the conclusions of this article will be made available by the authors, without undue reservation.

## Ethics statement

The animal study was reviewed and approved by Ethical Committee (76/2019, 2020/2019) at the Institute of Pharmacology, Polish Academy of Sciences, Krakow.

## Author contributions

All authors listed have made a substantial, direct, and intellectual contribution to the work and approved it for publication.
