# Supplementary material for: Combined treatment with Sigma1R and A2AR agonists fails to inhibit cocaine self-administration despite causing strong antagonistic accumbal A2AR-D2R complex interactions: the potential role of astrocytes
Source: Front Mol Neurosci. 2023 May 24;16:1106765. doi: 10.3389/fnmol.2023.1106765 (PMC10246738; doi:10.3389/fnmol.2023.1106765)

Supplementary Figure 1

Two-Way ANOVA analysis was performed to test the effects of treatment (vehicle vs OSU-6162) and drug use (cocaine vs yoked saline) on the average number of astrocytes, average number of branches per astrocyte, and branches length. The analysis showed significant interactions between the two factors for all three measures ($P<0.01$ for average number of astrocytes and branch length, and $P<0.05$ for average number of branches per astrocyte).

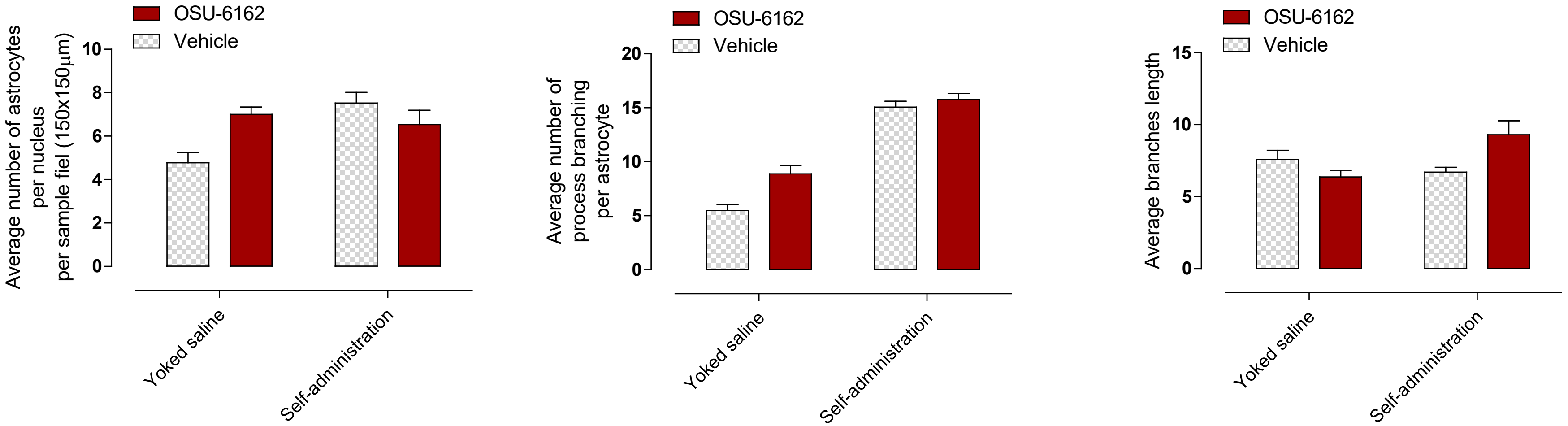

Source of Variation	P value summary	Significant?
Interaction	**	Yes
Column Factor	ns	No
Row Factor	*	Yes

Source of Variation	P value summary	Significant?
Interaction	*	Yes
Column Factor	**	Yes
Row Factor	***	Yes

Source of Variation	P value summary	Significant?
Interaction	**	Yes
Column Factor	ns	No
Row Factor	ns	No

Supplementary Figure 2

Proximity ligation assay (*in situ* PLA) was performed to assess the density of A2AR-D2R and D2R-Sigma1R clusters (red fluorescent puncta) in the nucleus accumbens (AcbSh) of the ventral striatum. The average number of PLA positive clusters (red) per nucleus (in blue) per sample field was used to determine the density of clusters. The results show a high density of A2AR-D2R and D2R-Sigma1R PLA positive clusters in the AcbSh, which was significantly different from the number of clusters in myelinated bundles of the crus cerebri (CC) and the anterior limb of the anterior commissure (aca), as well as negative controls (PLA experiments performed without one of the partner protomer, e.g., without anti-A2AR antibody and without anti-D2R antibody), which were considered as background values. Statistical analysis was performed by Mann Whitney U-test and by one way ANOVA followed by Tukey post hoc analysis, means ± SEM (n=4 rats).

Note:

(I) and (II) refer to experiments performed without one of the partner protomers, e.g., without anti-A2AR antibody and without anti-D2R antibody, respectively.

(III) and (IV) refer to A2AR-D2R and D2R-Sigma1R PLA positive clusters, respectively, found in the AcbSh.

(V) and (VIII) refer to myelinated bundles of the crus cerebri (CC), while (IV) and (VII) refer to the anterior limb of the anterior commissure (aca).

(IX) refers to the negative controls quantitation and the statistical analysis performed by Mann Whitney U-test, Mean and SEM, (n=4 rats)

(X) and (XI) refer to the analysis and comparison of the different brain regions (CC and aca, where considered as background). Statistical analyses were performed by one way ANOVA followed by Tukey post hoc analysis, , Mean and SEM, (n=4 rats).

Supplementary Figure 2

AcbSH: accumbens shell
aca: anterior commissure.

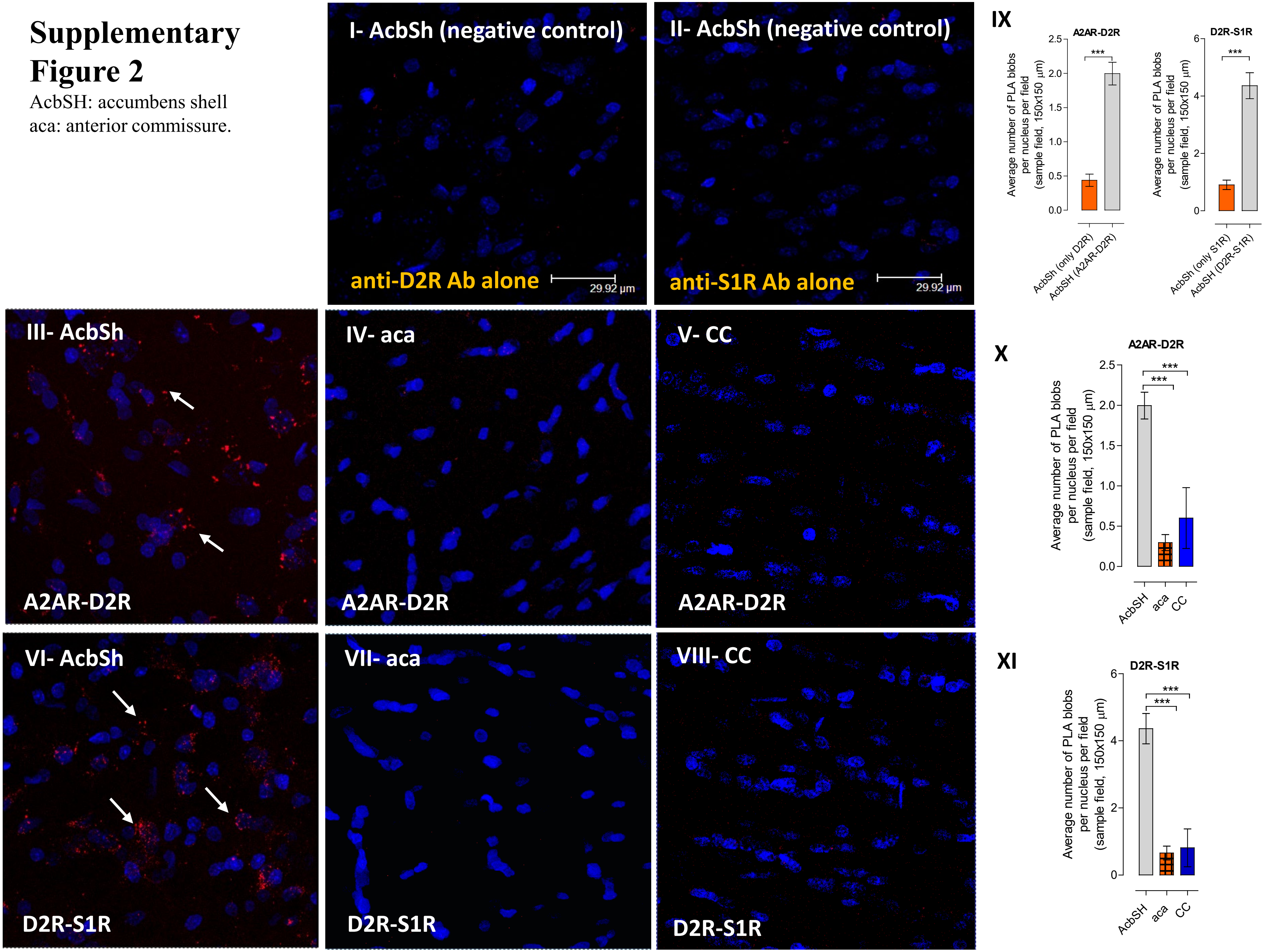

Supplement: Supplementary file 1 [file Data_Sheet_1.PDF]
